# Supplementary material for: Hyperactivation of Nrf2 in early tubular development induces nephrogenic diabetes insipidus
Source: Nat Commun. 2017 Feb 24;8:14577. doi: 10.1038/ncomms14577 (PMC5333130; doi:10.1038/ncomms14577)
Supplement: Supplementary Information — Supplementary figures and supplementary table. [file ncomms14577-s1.pdf]

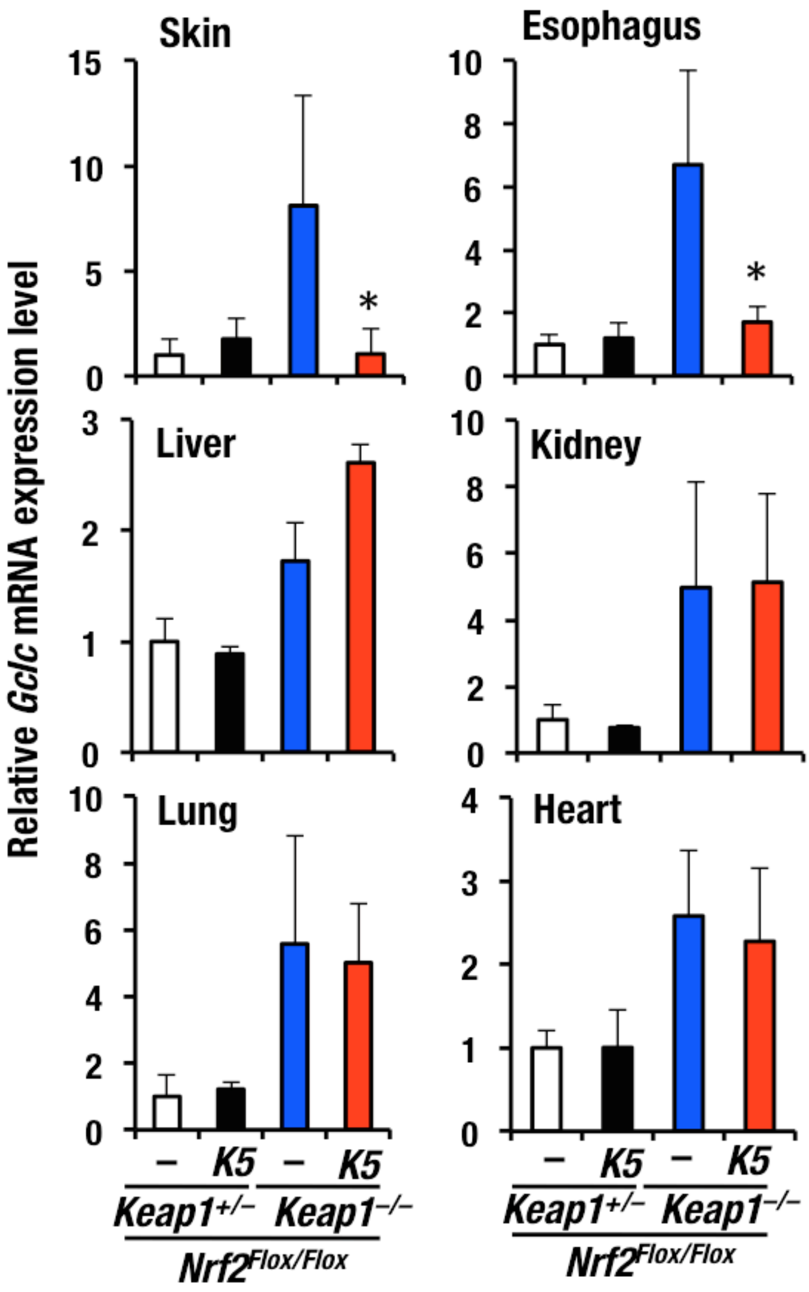

Supplementary Figure 1. Relative *Gclc* gene expression level normalized to *Hprt* expression in the esophagus, skin, liver, lung, kidney and heart of mice at 10 days of age. Data are the means ± SE (n=4). (*, $p<0.05$ compared to *Keap1*$^{-/-}$::*Nrf2*$^{Flox/Flox}$, unpaired *t*-test).

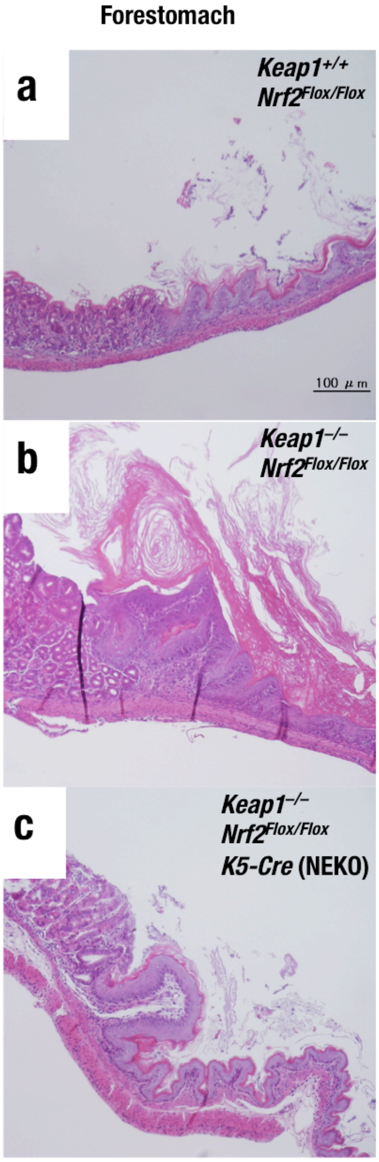

Supplementary Figure 2. (**a-c**) Representative images of HE staining of transverse sections of the forestomach from mice at 10 days of age. (**a**) *Keap1*$^{+/+}$*::Nrf2*$^{Flox/Flox}$ mouse forestomach, (**b**) *Keap1*$^{-/-}$*::Nrf2*$^{Flox/Flox}$ mouse forestomach, and (**c**) *Keap1*$^{-/-}$*::Nrf2*$^{Flox/Flox}$::K5-Cre mouse forestomach (n=3). Note that Nrf2 ablation rescued the hyper-keratinization phenotype of *Keap1*$^{-/-}$*::Nrf2*$^{Flox/Flox}$ mouse forestomach. Scale bar, 100 μm.

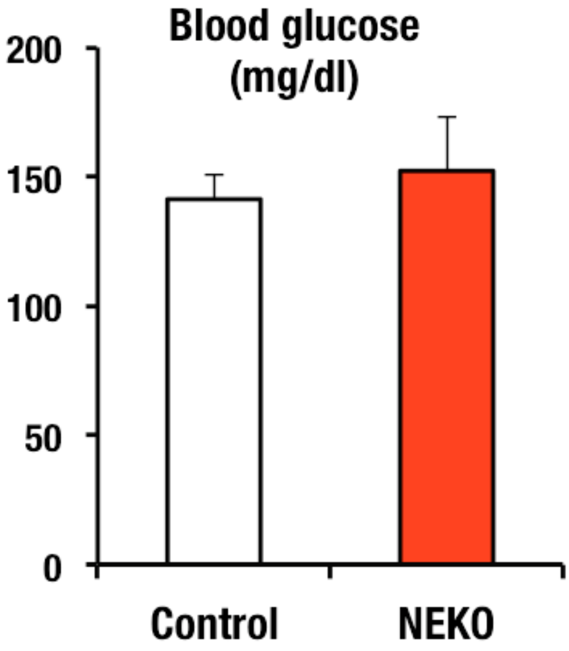

Supplementary Figure 3. Blood glucose levels of control and NEKO mice. Male mice of 8-12 weeks of age were utilized. Data are the means ± SE (n=5). Control genotype is described in Materials and Methods.

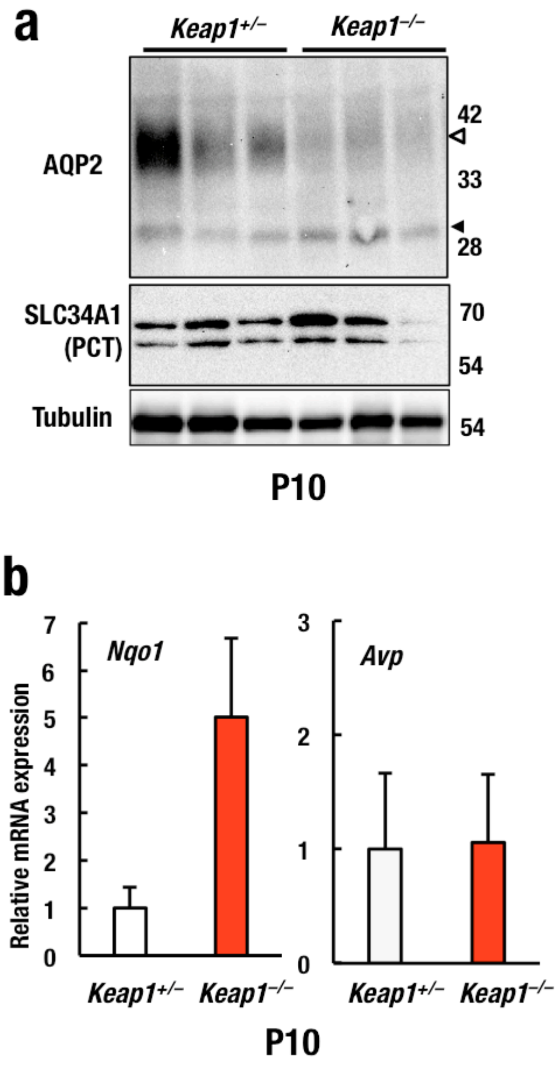

Supplementary Figure 4. (a) Immunoblot analysis of AQP2 and SLC34A1 proteins in kidneys of *Keap1*$^{+/-}$ and *Keap1*$^{-/-}$ mice at 10 days of age. Open and closed arrowheads indicate glycosylated and non-glycosylated forms of AQP2, respectively. The molecular weight standards are shown on the right. (**b**) Relative *Nqo1* and *Avp* gene expression levels in the hypothalamus of *Keap1*$^{+/-}$ and *Keap1*$^{-/-}$ mice at 10 days of age. Data are the means ± SE (n=6).

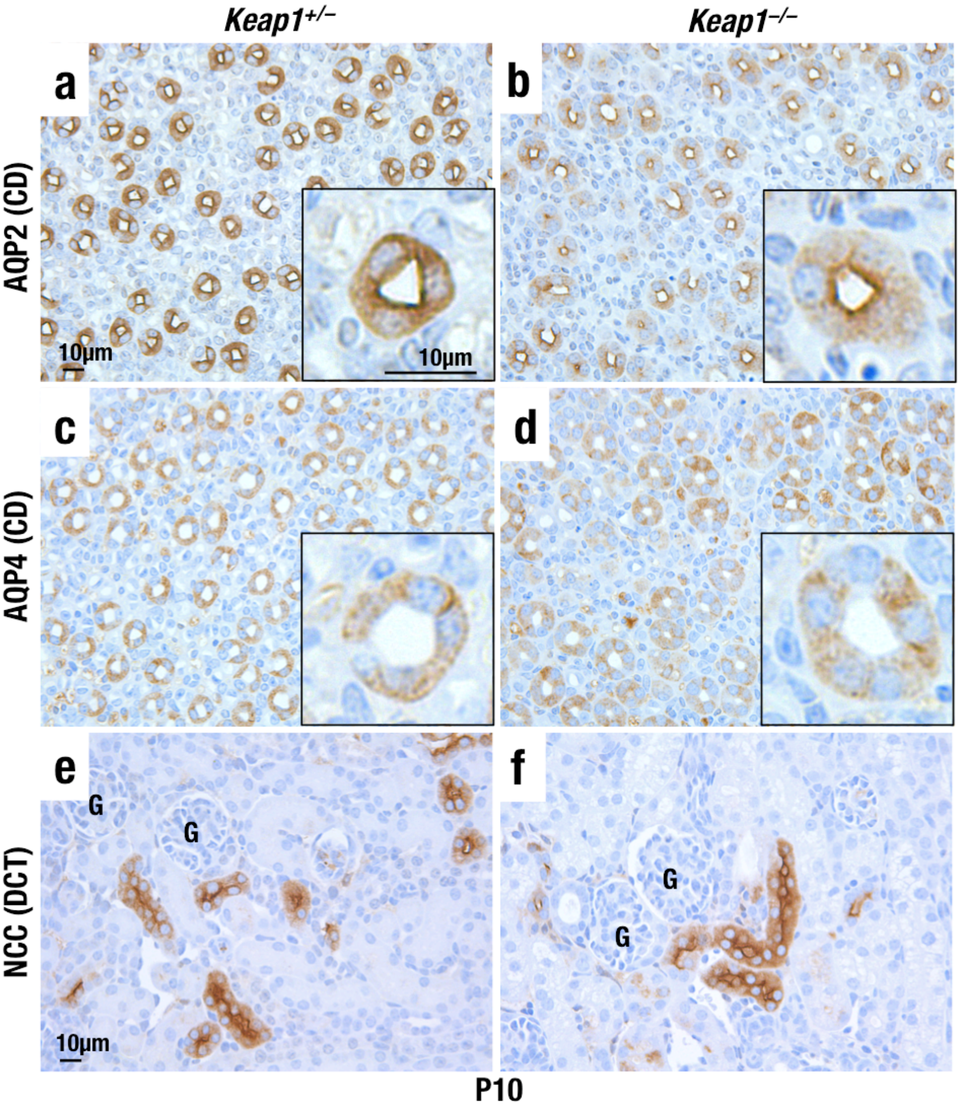

Supplementary Figure 5. Representative images of immunohistological staining for AQP2 (**a**,**b**), AQP4 (**c**,**d**) and NCC (**e**,**f**) of kidneys derived of *Keap1*$^{+/-}$ (**a**,**c**,**e**) and *Keap1*$^{-/-}$ (**b**,**d**,**f**) mice at 10 days of age (n=3). Scale bar, 10 µm. (**a-d**) Insets are higher magnification of collecting ducts. Scale bar, 10 µm. (**e**,**f**) Representative images of anti-NCC-stained renal cortex of *Keap1*$^{+/-}$ (**e**) and *Keap1*$^{-/-}$ (**f**) mice at 10 days of age. G, glomerulus. Scale bar, 10 µm. Note that accumulation of AQP2 in the apical region of the cells. CD, collecting ducts; DCT, distal convoluted tubules.

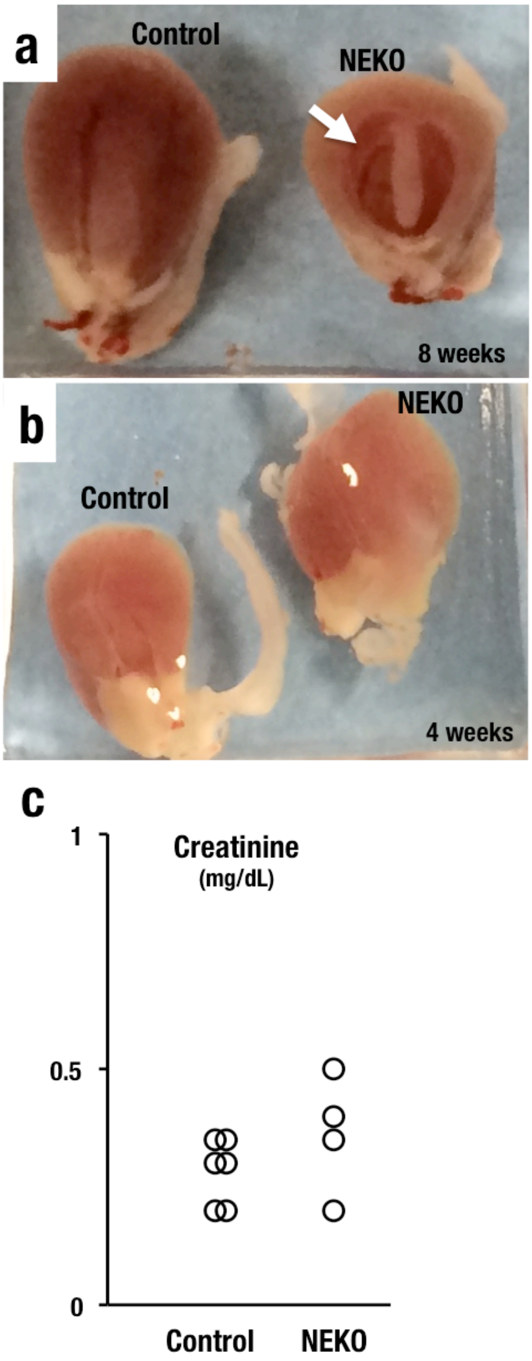

Supplementary Figure 6. (**a,b**) Coronal kidney sections of control and NEKO mice of 8 weeks (**a**) and 4 weeks (**b**) of age. (**c**) Plasma creatinine level of control and NEKO mice at 8-12 weeks of age.

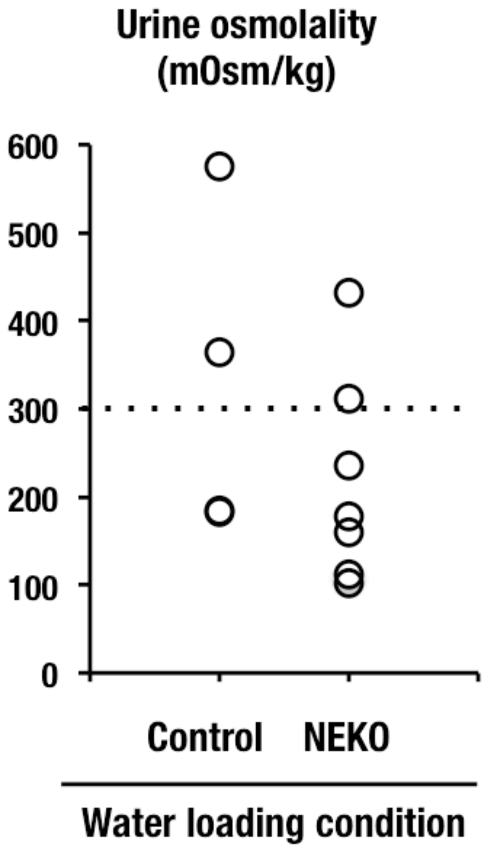

Supplementary Figure 7. Urine osmolality of control and NEKO mice at 8-12 weeks of age after challenging with water. Dotted line indicates the normal blood osmolality level.

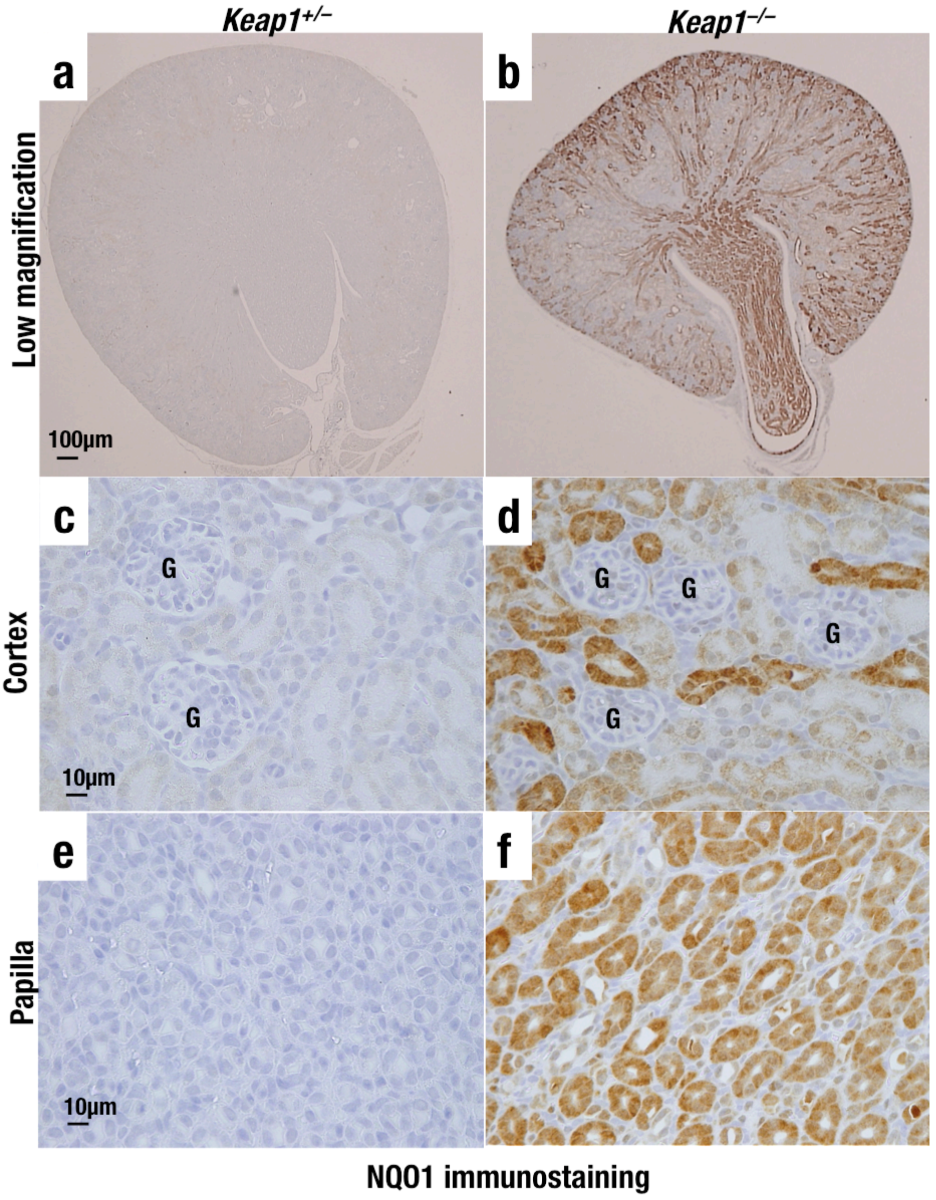

Supplementary Figure 8. NQO1 expression is highly induced in renal tubules and collecting ducts in *Keap1*-null mice. (**a-d**) Representative images of NQO1 immunostaining of kidneys from *Keap1*$^{+/–}$ (**a,c,e**) and *Keap1*$^{–/–}$ (**b,d,f**) mice at 10 days of age (n=3). (**a,b**) Lower magnification of coronal kidney sections. Scale bar, 100 μm. (**c-f**) Higher magnification of renal cortex (**c,d**) and papilla (**e,f**). G, glomerulus. Scale bar, 10 μm.

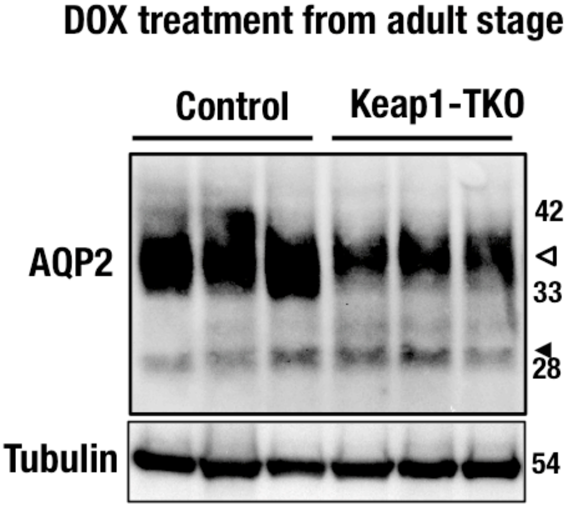

Supplementary Figure 9. Immunoblotting analysis of AQP2 protein in the kidney of control and Keap1-TKO mice treated with DOX during adult stage. Open and closed arrowheads indicate glycosylated and non-glycosylated forms of AQP2, respectively. The molecular weight standards are shown on the right.

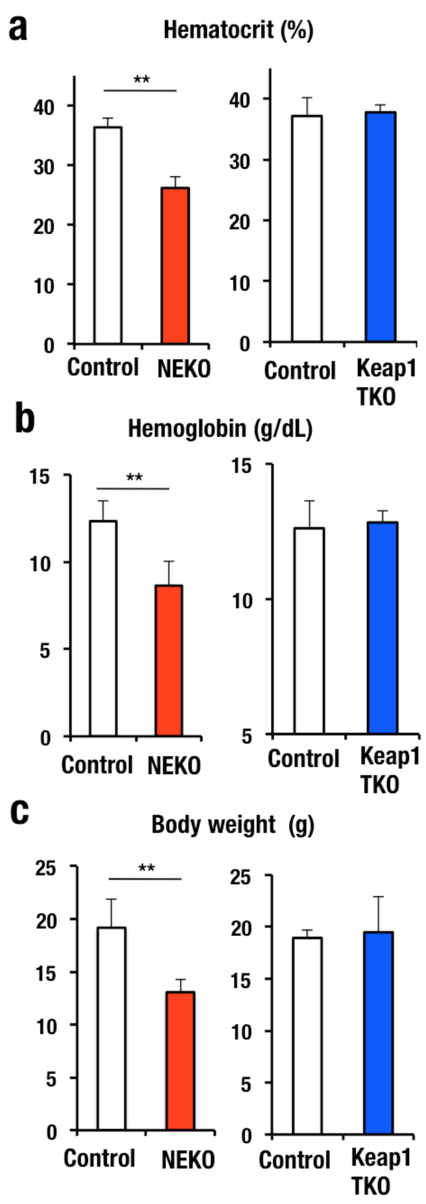

Supplementary Figure 10. (**a**,**b**) Hematocrit (**a**) and hemoglobin (**b**) of NEKO and Keap1-TKO mice at 8-12 weeks of age. Data are the means ± SE (n=6) (**, $p<0.01$, unpaired *t*-test). Keap1-TKO mice were treated with DOX from an embryonic stage. (**c**) Body weight of female NEKO and Keap1-TKO mice at 8 weeks of age. Data are the means ± SE (n=5) (**, $p<0.01$, unpaired *t*-test).

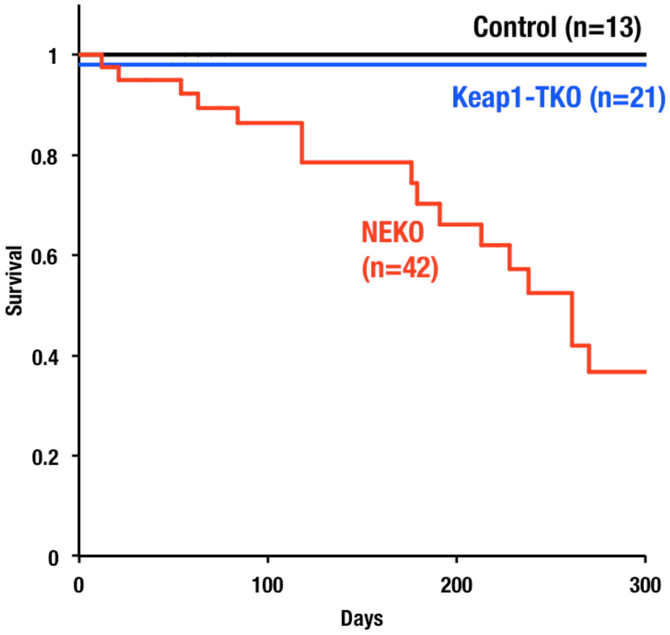

Supplementary Figure 11. Survival curve for control (n=13) and Keap1-TKO (n=21) mice treated with DOX from an embryonic stage. Survival curve of NEKO mice (n=42) in Figure 1 is also shown. Note that simple kidney tubule-specific knockout of Keap1 does not provoke lethality during this observation period.

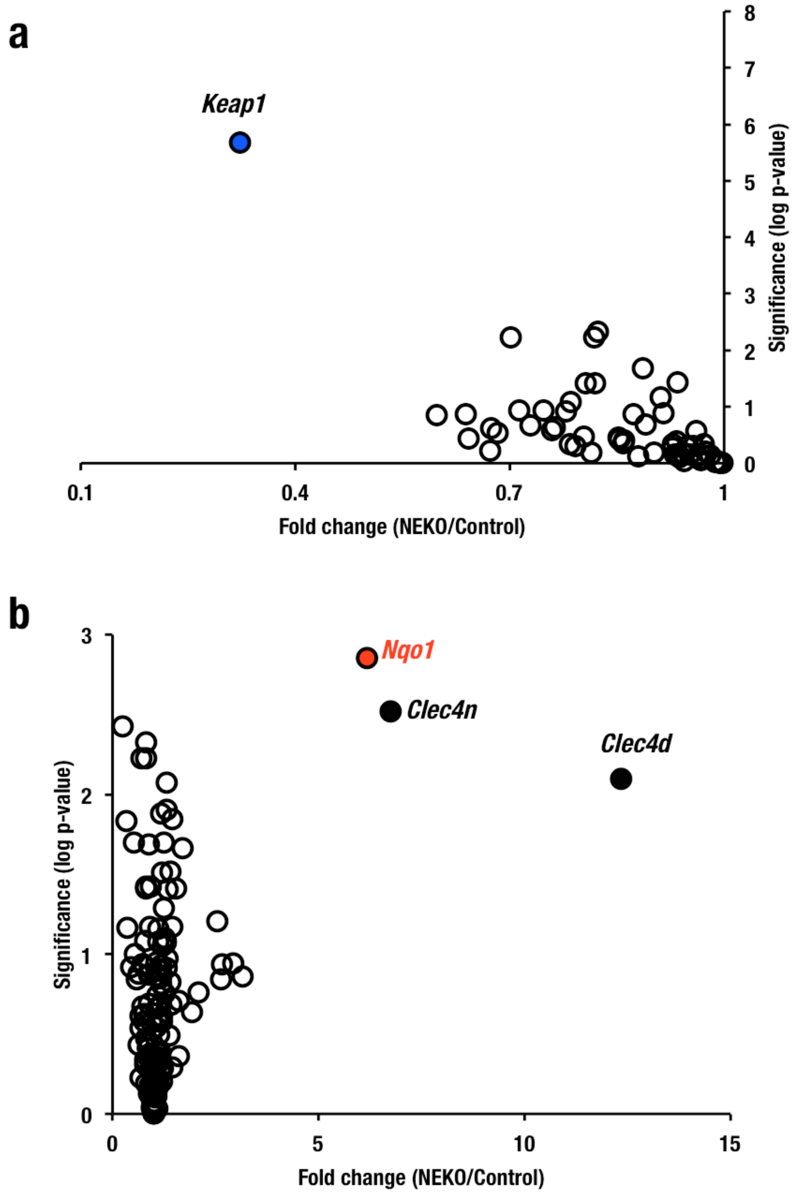

Supplementary Figure 12. Differentially-transcribed genes involving N-linked glycosylation and processing in the kidney of control and NEKO mice at 8 weeks of age. (**a**) Down-regulated genes in NEKO kidneys compared to controls. *Keap1* is shown as a positive control. (**b**) Up-regulated genes in NEKO kidneys compared to controls. *Nqo1* is shown as a positive control.

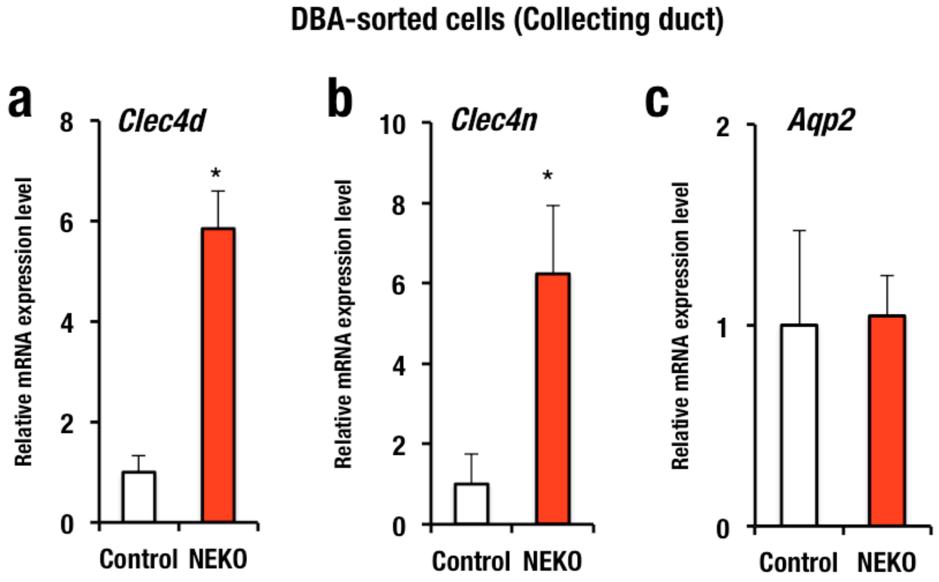

Supplementary Figure 13. Relative mRNA expression levels of *Clec4d, Clec4n* and *Aqp2* in DBA-sorted collecting ducts. Relative mRNA expression levels of *Clec4d* (**a**), *Clec4n* (**b**) and *Aqp2* (**c**) in control and NEKO mice at 8-12 weeks of age are normalized to *Hprt* gene expression in DBA-sorted collecting duct cells. Data are the means ± SE (n=3). (*, $p<0.05$ compared to control, unpaired *t*-test).

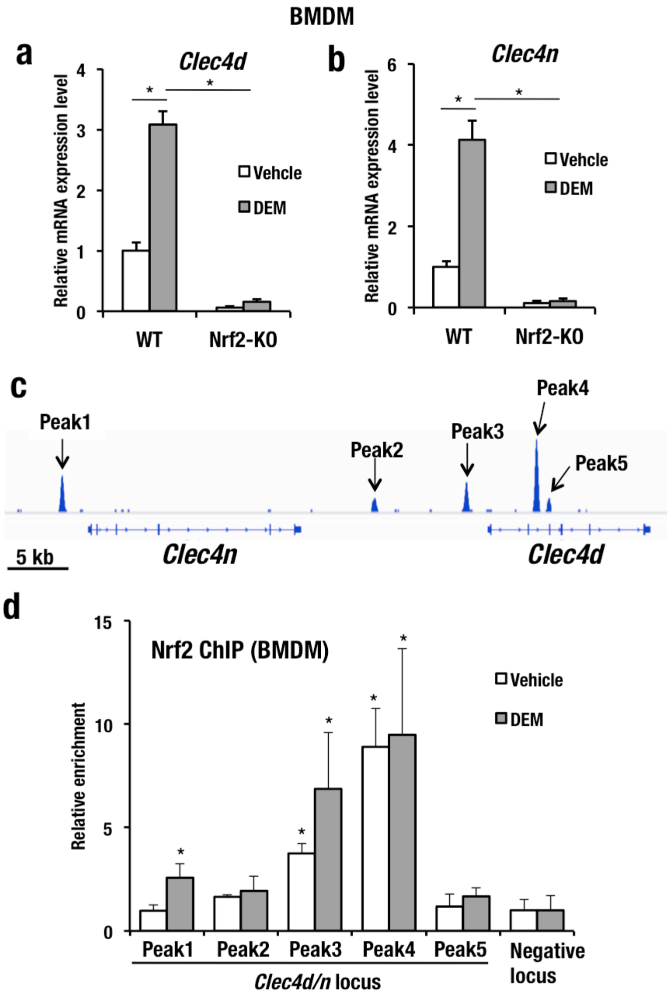

Supplementary Figure 14. Relative mRNA expression level of *Clec4d* (**a**) and *Clec4n* (**b**) normalized to *Gapdh* gene expression in bone marrow derived macrophages (BMDMs) from wild-type and *Nrf2*-null mice with or without 100-µM DEM treatment for 6 hours. Data are the means ± SE (n=3) (*, p<0.05, unpaired *t*-test). (**c**) Nrf2 binding to the proximity of the *Clec4d* and *Clec4n* genes in BMDMs from Nrf2 ChIP-seq analyses data[24]. (**d**) Nrf2 ChIP-qPCR analyses of BMDMs treated with or without 100-µM DEM for 4 hours. Data are the means ± SE (n=3) (*, p<0.05 compared to negative locus, unpaired *t*-test).

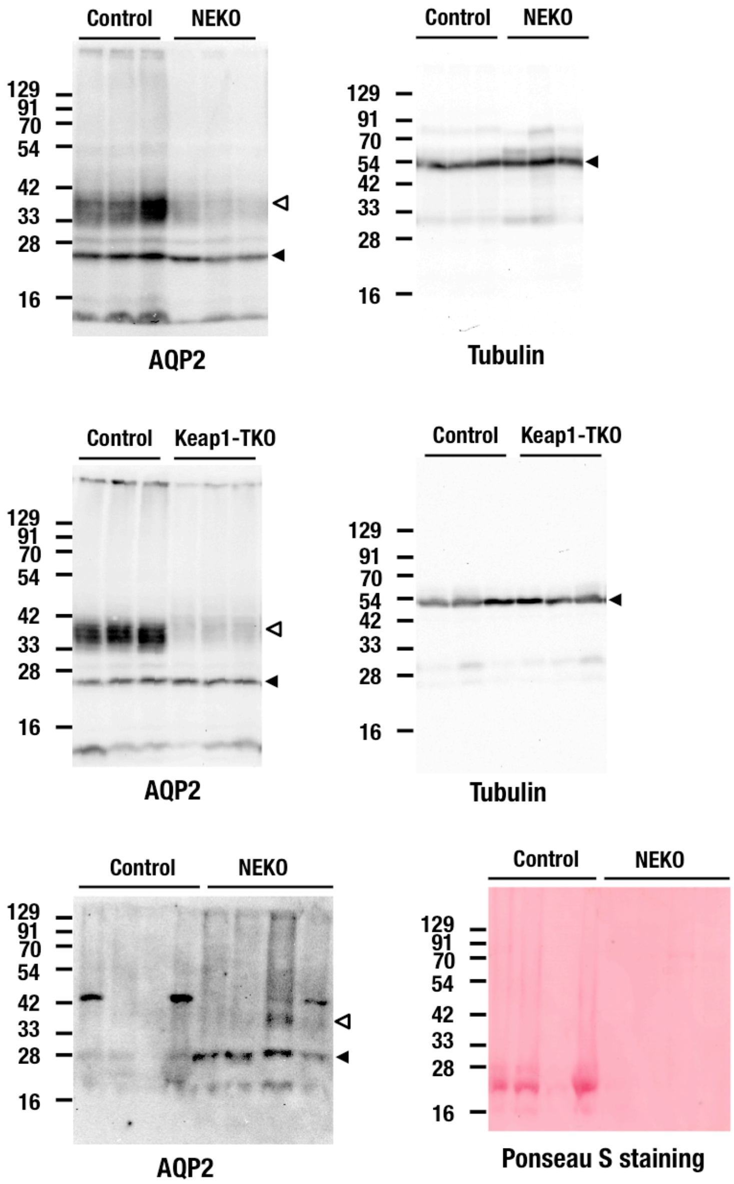

Supplementary Figure 15. Full blot and gel images for Figures 3d, 7e and 8a.

Supplementary Table 1

List of primer and probe sequences used in this study.

Assay	Gene	Sequence
RT-PCR (TaqMan)	*Nqo1*	Sense AGCTGGAAGCTGCAGACCTG<br>Antisense CCTTTCAGAATGGCTGGCA<br>Probe ATTTCAGTTCCCATTGCAGTGGTTTGGG
	Hprt	Sense CTGGTGAAAAGGACCTCTCG<br>Antisense TGAAGTACTCATTATAGTCAAGGG<br>Probe ATCCAACAAAGTCTGGCCTGTATCCAAC
	Keap1	Sense GATCGGCTGCACTGAACTG<br>Antisense GGACTCGCAGCGTACGTT<br>Probe CTGGCCACGCTCATCAGCCG
RT-PCR (SYBR-Green)	*Avp*	Sense GCTCTCCGCTTGTTTCCTGA<br>Antisense TGGGCAGTTCTGGAAGTAGCA
	Aqp2	Sense CAGCTCGAAGGAAGGAGACA<br>Antisense GCATTGGCACCCTGGTTCA
	Clec4d	Sense GATGAGCAGTCATCTGGTGAC<br>Antisense TTCCCAGAATACCGTGTGTG
	Clec4n	Sense CTGCCCAAATCACTGGAAGT<br>Antisense ATCCGAAAGACCCAGGAAGT
ChIP-qPCR	*Clec4 Peak 1*	Sense GGGGAATGTTTTCTCTTCCTG<br>Antisense AGGCATTACCAGCTGTTTCC
	Clec4 Peak 2	Sense ACCTTCCTCTTTGCTGGTGA<br>Antisense CTGCTCATGGCAAGATGGTA
	Clec4 Peak 3	Sense GGAGCCTGGGACCACTATTT<br>Antisense GCACATCATTGTGGAAGAGAC
	Clec4 Peak 4	Sense TCGGGAAAGGTGTTTTGTTG<br>Antisense GACACTGGGTGTGACATGGT
	Clec4 Peak 5	Sense TTTCATTGAATTTATCTGGGATCA<br>Antisense AGTCCCCACACCCTCTTTCT
	Clec4 Negative locus	Sense TAGAGTGACATTGTGGCGCA<br>Antisense AGGAGCCAGAGACAGCCTAA
